# Comparative Analysis of Chloroplast Genomes for the Genus *Manglietia* Blume (Magnoliaceae): Molecular Structure and Phylogenetic Evolution

**DOI:** 10.3390/genes15040406

**Published:** 2024-03-26

**Authors:** Tingzhang Li, Shuangyu Zhang, Yunfei Deng, Yuling Li

**Affiliations:** 1 College of Forestry and Landscape Architecture, South China Agricultural University, Guangzhou 510642, China; litingzhang666@stu.scau.edu.cn (T.L.); zhangshuangyu@stu.scau.edu.cn (S.Z.); 2 State Key Laboratory of Plant Diversity and Specialty Crops, South China Botanical Garden, Chinese Academy of Sciences, Guangzhou 510650, China; 3 Key Laboratory of National Forestry and Grassland Administration on Plant Conservation and Utilization in Southern China, Guangzhou 510650, China; 4 Key Laboratory of Plant Resources Conservation and Sustainable Utilization, South China Botanical Garden, Chinese Academy of Sciences, Guangzhou 510650, China

**Keywords:** *Manglietia*, chloroplast genome, phylogenetic, molecular marker, interspecific relationship

## Abstract

*Manglietia* Blume, belonging to the Magnoliaceae family and mainly distributed in tropical and subtropical regions of Asia, has great scientific and economic value. In this study, we employed next-generation sequencing followed by de novo assembly to investigate the adaptive evolution of *Manglietia* using plastid genetic information. We newly sequenced the complete or nearly complete plastomes of four *Manglietia* species (*Manglietia aromatica*, *Manglietia calcarea*, *Manglietia kwangtungensis*, and *Manglietia glauca*) and conducted comparative analysis with seventeen published plastomes to examine the evolutionary pattern within this genus. The plastomes of these five newly sequenced *Manglietia* species range from 157,093 bp (*M. calcarea*2) to 160,493 bp (*M. kwangtungensis*), all exhibiting circular structures when mapped. Nucleotide diversity was observed across the plastomes, leading us to identify 13 mutational hotspot regions, comprising eight intergenic spacer regions and five gene regions. Our phylogenetic analyses based on 77 protein-coding genes generated phylogenetic relationships with high support and resolution for *Manglietia.* This genus can be divided into three clades, and the previously proposed infrageneric classifications are not supported by our studies. Furthermore, the close affinity between *M. aromatica* and *M. calcarea* is supported by the present work, and further studies are necessary to conclude the taxonomic treatment for the latter. These results provide resources for the comparative plastome, breeding, and plastid genetic engineering of Magnoliaceae and flowering plants.

## 1. Introduction

Magnoliaceae Juss. comprises evergreen and deciduous trees or shrubs with showy flowers, boasting a rich diversity of over 300 species primarily found in Southeast Asia and the Americas. It is recognized as one of the most primitive and endangered groups of angiosperms [1,2], holding significant value for investigating the origin and phylogeny of flowering plants. Due to the reticulate evolution of morphological characteristics [3], there has been long debate over the classification of Magnoliaceae regarding the delimitation of tribes, genera, and sections. Currently, the most prominent classification systems for Magnoliaceae have been proposed by Dandy [4,5,6], Law [7,8], Nooteboom [9], and Figlar and Nooteboom [10]. Dandy [4,5,6] recognized twelve genera in two tribes, *Liriodendron* Linn. in the tribe Liriodendreae and others in the tribe Magnolieae. Law [7,8] upgraded Dandy’s two tribes to the rank of subfamily, i.e., Liriodendroideae and Magnolioideae, and further divided Magnolioideae into two tribes and five subtribes, recognizing 16 genera. Nooteboom [9] accepted Law’s infrafamilial classification but he merged some genera into others. However, Figlar and Nooteboom [10] proposed a quite different classification by merging all genera except *Liriodendron* into a single expanded genus, *Magnolia* Linn., which was further divided into three subgenera and twelve sections. In the *Flora of China* [11], Xia classified the Chinese species of Magnoliaceae into thirteen genera, including three new genera, while Nooteboom, another co-author of the account of the family for the flora, accepted only two genera, *Liriodendron* and *Magnolia*, following the treatment of Figlar and Nooteboom [10]. Furthermore, many molecular studies of the phylogeny of the family Magnoliaceae were performed using chloroplast [12,13,14,15,16,17,18,19,20,21,22,23] and nuclear datasets [24]. The phylogenetic results based on cpDNA [17,18,21] or nuclear data [24] indicated that 12–16 major clades were found to be monophyletic within Magnoliaceae. Accordingly, Nie et al. [24] proposed an updated classification of the genus *Magnolia* into 15 sections, and each section was treated as independent genus by Xia [11] and Sima and Lu [3]. Although many phylogenetic studies have been conducted to enhance our understanding of the phylogenetic evolution in the family, the deeper relationships within each clade, such as *Manglietia* Blume, remains poorly resolved due to limited taxon sampling. The classification of Xia is widely accepted in Asia, especially in China [25,26,27,28]. Here, we accepted to the generic delimitation, in a narrow sense, in the classification of the family Magnoliaceae by Xia [11] and Sima and Lu [3], which treats all sections of Nie et al. [24] as independent genera.

*Manglietia* Blume, established by Blume [29], comprises approximately 40 species primarily distributed in tropical and subtropical regions of Asia, with a concentration of to 27 or 29 species in Southern China [11]. This genus is characterized by its evergreen habit (except *Manglietia decidua* Q. Y. Zheng), stipules adnate to the petioles, and the presence of four or more ovules per carpel [30]. It was later treated as a section of *Magnolia* by some scholars [10,21,31]. It is generally considered closely allied to the genera *Magnolia* and *Manglietiastrum* Law. based on its morphological characteristics [32,33,34,35]. Furthermore, the infrageneric classification of *Manglietia* is still unclear. Tiep [36] was the first one to establish the infrageneric classification of *Manglietia*, and recognized two sections, i.e., sect. *Olivera*, with its style shorter than half of the carpel length, and sect. *Manglietia*, with its style longer than half of the carpel length. But this classification has never been followed or adopted by later authors. Zheng [37] merged the monotypic genus *Sinomanglietia* Z.X. Yu, which contains a single species (*Simarouba glauca* Z.X. Yu) with deciduous habits, into *Manglietia* and treated it as sect. *Deciduae* Q. Y. Zheng [38,39], while placing all other species with evergreen habits into sect. *Manglietia*. However, all previous molecular studies indicated that *Manglietia* was strongly supported to be monophyletic [17,18,20,22], and it was recognized as an independent genus for long time in Asian flora and checklists [8,11]. Several phylogenetic analyses of *Manglietia* have been carried out, but no texts have been produced on the infrageneric classification within *Manglietia*. Thus, the phylogenetic relationships within *Manglietia* also remain unresolved, and further studies are necessary.

Since the sequencing of the first chloroplast genome (plastome) in 1986 [40], there has been rapid progress in high-throughput sequencing technology, resulting in the continuous sequencing of the numerous plant plastomes [41,42]. The National Center for Biotechnology Information (NCBI) organelle genome database currently holds more than 8600 plant plastomes, with the majority of them sequenced in the past four years [43]. In contrast to nuclear genomes, plastomes typically exhibit a conserved structure and a relatively lower rate of nucleotide substitution [44,45]. They have been extensively utilized for resolving phylogenetic relationships among plant lineages and investigating chloroplast genome evolution, such as in Dennstaedtiaceae Lotsy. [46] and *Magnolia* [47]. These investigations significantly advanced our understanding of plant evolutionary relationships. However, the plastomes of many species of *Manglietia* are only published in the form of data, and scientific problems such as structural variation and evolutionary relationships among lineages have not been discussed [23,48]. Given their significant scientific and economic value, further exploration employing chloroplast genomes would provide crucial insights into the systematics and evolution of the family Magnoliaceae [11].

Traditionally based on morphological characteristics alone, species delimitation often fails to distinguish recently diverged species, resulting in cryptic species complexes [49]. These species can be derived from isolation differentiation [50], hybridization, and polyploidization [51]. The availability of high-throughput sequencing technology has made obtaining plastome sequences more feasible. Compared to nuclear genomes and mitochondrial genomes, plastomes possess a small size, a low rate of nucleotide substitution, single-parental inheritance, and a haploid nature. These characteristics make plastomes an excellent choice for analyzing nucleotide diversity and reconstructing phylogenies among closely related species, particularly among polyploid taxa [52,53]. Numerous studies have utilized plastome data to resolve species classification, elucidate phylogenetic relationships among land plants, and conduct comparative analyses of chloroplast genomes [54,55,56]. Notably, comparative analysis based on plastome data provides a more comprehensive understanding of species evolution and phylogenetic relationships compared to limited DNA fragments [57]. Among *Manglietia* species, *Manglietia calcarea* X. H. Song is a rare and endangered and extremely small population distributed in the neighboring region of N Guangxi and S Guizhou [58,59]. *M. calcarea* has a high calcium requirement, a narrow ecological environmental range, and a small population, its distribution areas are relatively remote, and it is endemic to Guizhou and Guangxi [59]. *M. calcarea* was described by Song [60] as originating from the limestone areas in Libo County, Guizhou Province, and its taxonomic status has been controversial. Chen and Nooteboom [35] considered *M. calcarea* to be similar to *Manglietia fordiana* Oliv. and treated it as *M. fordiana* var. *calcarea*. *M. calcarea* was neglected in the *Flora Reipublicae Popularis Sinica* [30] and reinstated in *Flora of China* [11]. Sima et al. [59] compared *M. calcarea* with *M. aromatica* Dandy, and *M. fordiana* based on 13 morphological characteristics, and concluded that it is very similar to *M. aromatica*; they treated it as a variety *M. aromatica* var. *calcarea*. However, this result was based only on morphological characteristics; the classification status of *M. calcarea* needs be evaluated. To better understand the classification status of *M. calcarea* and its relationship with *Manglietia*, it is imperative to identify genetic discrepancies within the major clade of *Manglietia*.

In this research, we have assembled and annotated the five plastomes of four *Manglietia* species. The present work aims to (1) investigate the genetic variation within the *Manglietia* plastome; (2) characterize plastomes structure, sequence divergence, mutation hotspot regions, and repeat regions; (3) evaluate the phylogenetic relationships within *Manglietia*; and (4) clarify the classification relationships of related genera and the specular species *M. calcarea*.

## 2. Materials and Methods

### 2.1. Plant Materials and DNA Extraction and Sequencing

The fresh leaves of five samples representing four species of *Manglietia* (*M. aromatica*, *M. calcarea*, *M. glauca* Blume, and *M. kwangtungensis* Dandy) were collected from the provinces of Guangxi, Guizhou, and Guangdong, respectively. Among them, two samples of *M. calcarea* from different locations were added for a comparative analysis of chloroplast genomes on the population level to better understand the classification relationships among the closely related species of *M. calcarea*. The voucher specimens were deposited at the Herbarium of South China Agricultural University (CANT) and the Herbarium of South China Botanical Garden, Chinese Academy of Sciences (IBSC) (Table S1). Total genomic DNA was extracted from fresh, young leaves using the Plant Genomic DNA Kit (Tiangen, Beijing, China) following the manufacturer’s protocol. Once the sample genomic DNA passed quality assessment, short-insert (500 bp) paired-end (PE) libraries were sequenced by the Beijing Genomics Institute (Shenzhen, China) using the Illumina HiSeq 2500 platform with a read length of 150 bp. Each species generated a minimum of 5 Gb clean data.

### 2.2. Plastid Genome Assembly, Annotation, and Comparison

The paired-end reads from the clean data were filtered and assembled into contigs using the GetOrganelle software [61]. Subsequently, the assembled plastomes were visually inspected in Bandage [62] software followed by manual editing if necessary in Geneious v11.1.5 software; then, complete plastomes were obtained for each sample.

The plastomes were annotated using Plastid Genome Annotator (PGA) software [63], with the reference plastome of *M. aromatica*2 (NC_037000.1). Manual inspection and editing were carried out in Geneious v11.1.5 [64] as needed. Furthermore, tRNA genes were annotated in Geneious v11.1.5 using the genome of *M*. *aromatica*2, with sequence similarity threshold set at >75%. Finally, five high-quality, complete or nearly complete chloroplast genomes were obtained. Organellar Genome DRAW (OGDRAW) v1.3.1 was used to visualize the structural features of the four species [65].

### 2.3. Plastome Comparison and Sequence Divergence Analysis

Seventeen whole plastomes of *Manglietia* (*M. aromatica*2, *M. calcarea*3, *M. conifera* Dandy, *M. glaucifolia* Y. W. Law & Y. F. Wu, *M. dandyi* (Gagnep.) Dandy, *M. zhengyiana* N. H. Xia, *M. lucida* B. L. Chen & S. C. Yang, *M. patungensis* Hu, *M. crassipes* Y. W. Law, *M. ventii* N. V. Tiep, *M. fordiana*, *M. insignis* (Wall.) Blume, *M. duclouxii* Finet & Gagnep., *M. hookeri* Cubitt & W. W. Sm., *M. grandi*s Hu & W. C. Cheng, *M. obovalifolia* C. Y. Wu & Y. W. Law, and *M. decidua*) were downloaded from NCBI. Together with five newly sequenced plastomes, this dataset provided the possibility of conducting comparative analysis among relatives. Mauve alignment was employed for analyzing plastomes’ DNA rearrangement across all 22 *Manglietia* sequences [66]. The online tool IRscope (https://irscope.shinyapps.io/irapp/ (accessed on 24 February 2023)) was utilized to compare the junction regions among the 22 sequences that connect the IR, SSC, and LSC [67]. Additionally, the sequence divergence of 22 *Manglietia* plastomes was investigated using the program mVISTA [68], with LAGAN and *M. aromatica*2 as references, to demonstrate inter- and intraspecific variations. Nucleotide diversity was also assessed by DnaSP v6.12.03 (DNA Sequences Polymorphism) software with a sliding window strategy [69], where the step size was set to 200 bp and a window length of 600 bp was employed [70].

The codon usage pattern of protein-coding genes in the 22 *Manglietia* plastomes was estimated using CodonW v.1.4.2. (https://sourceforge.net/projects/codonw/ (accessed on 1 March 2023)). The relative synonymous codon usage (RSCU) values and the effective number of codons (ENC) were determined to quantify the extent of the codon usage bias for each genome by applying published equations for RSCU calculation [71]. Subsequently, the TBtools HeatMap function [72] visualized the RSCU values across the 22 *Manglietia* plastomes, while the ENC values indicated each individual gene’s codon bias within a range from 20 to 61; lower ENC values denoted higher codon bias levels observed in specific genes [73]. The computation of overall GC content and the individual GC content at the first, second, and third codon positions (GC1, GC2, and GC3, respectively) were calculated utilizing EMBOSS [74] online software (http://emboss.toulouse.inra.fr/cgi-bin/emboss/ (accessed on 1 March 2023)).

### 2.4. Repeat Sequence Analysis

The REPuter [75] online software (https://bibiserv.cebitec.uni-bielefeld.de/reputer/ (accessed on 26 February 2023)) facilitated the identification of repeat sequences, including palindromic repeats, direct repeats, and reverse repeats, under specified parameters: The maximum size of repeat sequences that were computed was limited to 50, while the minimum size and Hamming distance were set at 30 and 3. Tandem repeat sequences were identified through Tandem Repeats Finder [76] employing alignment parameters such as match = 2, mismatch = 7, and indels = 7. Repeats satisfying the conditions of a minimum alignment score of 80, a maximum period size of 500 bp, and a maximum TR array size of 2 million were considered. Furthermore, simple sequence repeat (SSR) detection utilized the Perl script MISA (MIcroSAtellite identification tool), with a threshold of mono-, di-, tri-, tetra-, penta-, and hexanucleotides, respectively [77].

### 2.5. Analysis of Substitution Rate

In this study, we employed the KaKs_calcaulator [78] to calculate nonsynonymous (Ka) and synonymous substitution rates (Ks), as well as the Ka/Ks ratio, in order to identify gene divergence change within the 22 *Manglietia* plastomes. To minimize errors, we screened the protein-coding gene (CDS) sequences of these plastomes using specific criteria: each CDS sequence should have a total number of bases that is a multiple of 3 and a length > 300 bp. Subsequently, we retained 51 CDS sequences for further analysis. Pairwise comparisons among the 22 plastomes resulted in a total of 231 sequence pairs. The genetic code was set as the “Bacterial and Plant Plastid Code” with the calculation method “YN”. When there were no substitutions or perfect matches in the alignment, the Ks value was set to 0; in such cases, the Ka/Ks value was reported to be “NA” and replaced with 0 in the results.

### 2.6. Dataset Generation and Phylogenetic Analyses

A total of 57 complete Magnoliaceae family plastomes belonging to all sections recognized by Wang et al. [21] were obtained from the NCBI GenBank database. *Liriodendron tulipifera* Linn. and *Liriodendron chinense* (Hemsl.) Sargent were used as outgroups. For the phylogenetic analysis, the distribution of the 57 sequences among different genera in Magnolioideae were as follows: *Manglietia* (22), *Houpoea* N. H. Xia & C. Y. Wu (3), *Aromadendron* Blume (2), *Magnolia* (3), *Michelia* Linn. (3), *Oyama* (Nakai) N. H. Xia & C. Y. Wu (2), *Pachylarnax* Dandy (4), *Kmeria* (Pierre) Dandy (1), *Metamagnolia* Sima & S. G. Lu (3), *Paramagnolia* Sima & S. G. Lu (2), *Dugandiodendron* Lozano (5), *Yulania* Spach (4), *Lirianthe* Spach (2), and *Talauma* Juss. (1).

All the annotated filest of the nucleotide sequences of the protein-coding genes (CDS) in GenBank format were extracted using Geneious v11.1.5 software and manually corrected if necessary. CDS alignment was performed using Muscle v3.8.31 [79] software and manually adjusted when required. Loci covering less than 55% of species were removed to minimize reliance on loci with limited information or present in relatively few species, resulting in obtaining a final set of 77 CDS sequences from 59 plastomes for subsequent analysis. The script “concatenate_fasta.py” (available at https://github.com/Kinggerm/PersonalUtilities/ (accessed on 31 October 2023)) was utilized to merge locus alignments and generate CDS datasets. Furthermore, Gblocks v0.91b was used with strict exclusion criteria (-b5 = *n*) to generate the CDS_GB datasets [80].

The gene trees for the CDS_GB datasets were inferred using maximum likelihood (ML) and Bayesian inference (BI) methods. For phylogenetic inference, the ML tree was constructed with IQ-TREE v2.0.3, employing 1000 bootstrap replicates. The optimal nucleotide substitution model was determined using parameters set as “-MFP” in IQ-TREE [81]. Bayesian inference (BI) was performed with Mrbayes v3.2 [82]. Markov chain Monte Carlo (MCMC) analysis was run for 1,000,000 generations. The ML and BI trees were visualized using FigTree v1.4.3 (http://tree.bio.ed.ac.uk/software/figtree/ (accessed on 20 February 2024)).

## 3. Results

### 3.1. Features of Manglietia Plastome

**Figure 1:**
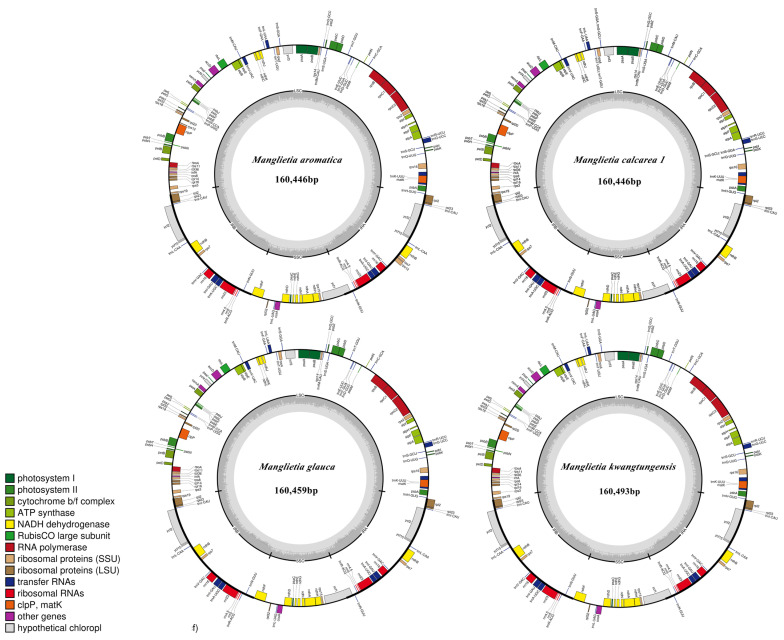
Four newly sequenced gene maps of *Manglietia* plastomes were generated using the OGDRAW online platform. Clockwise transcription is observed for genes located inside the circle, while counterclockwise transcription occurs for those outside. Color-coded representation distinguishes genes with different functions. Large single-copy region (LSC), small single-copy region (SSC) and inverted repeat region (IR) genes are indicated. Additionally, GC content is depicted by a darker shade in the inner circle, whereas AT content is represented by a lighter gray shade.

All chloroplast genomes exhibited a double-stranded circular quadripartite structure in these species’ plastomes (Figure 1). The 22 plastomes ranged in size from 157,093 bp in *M. calcarea*2 to 160,493 bp in *M. kwangtungensis* (Table 1). All complete or nearly complete plastomes comprised a large single-copy region (LSC 87,959 bp–88,791 bp), a small single-copy region (SSC 18,741 bp–19,030 bp), and a pair of inverted repeat regions (IR 24,991 bp–26,782 bp). For each assembled chloroplast genome, 110–113 genes were annotated, including 79 protein-coding genes, 28–30 tRNA genes, and 3–4 rRNA genes (Table 1 and Table 2). The overall GC content of these plastomes was similar, all of which were 39.30%, except *M. calcarea*2 (39.00%), and varied within the LSC, SSC, and IR regions. The GC content in the IR region (42.50%–43.20%) was higher than that in LSC (37.90%–38.00%) and SSC (34.20%–34.30%) regions (Table 1). In all species’ plastomes examined, the *ycf1* gene extended from the IRa into the SSC region while leaving a truncated copy at the junction of IRb/SSC. The gene *rps12* underwent trans-splicing across two regions: its 5′ end exon resided in the LSC region, whereas its intron and 3′ end exon was located within the IR region. The plastid genome sequences obtained have been submitted to GenBank (Accession Nos. PP386157–PP386161).

### 3.2. Comparative Genomic Analysis of Manglietia Species

**Figure 2:**
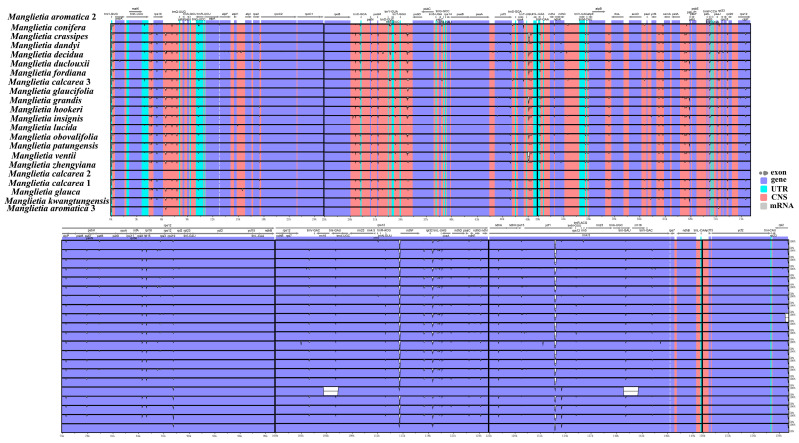
Comparison of twenty-two plastomes from *Manglietia* using mVISTA program, with the annotation of *M. aromatica*2 as a reference. Pink regions indicate conserved non-coding areas, purple represents conserved exons, and white regions denote more variable sites. The vertical scale illustrates percent identity ranging from 50% to 100%.

**Figure 3:**
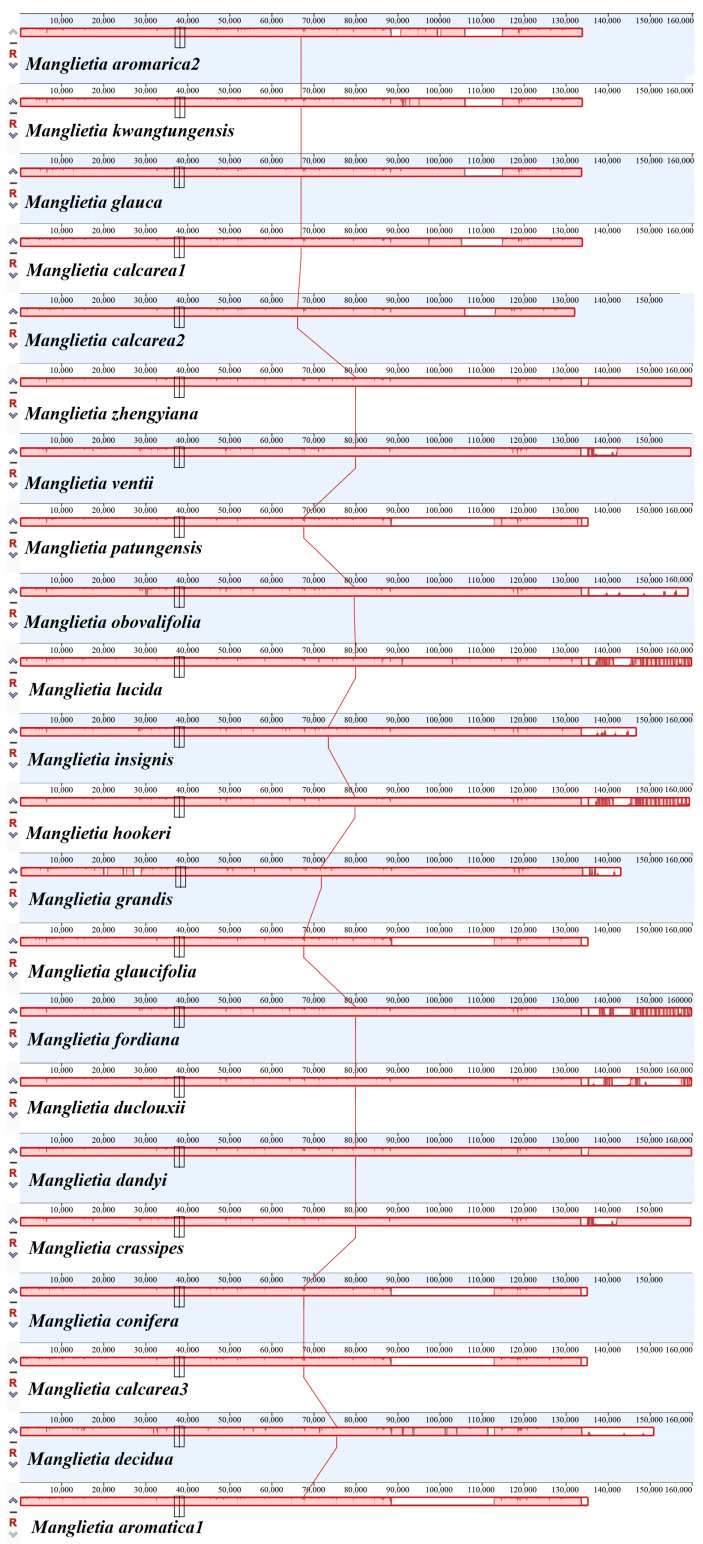
Mauve alignment of twenty-two plastomes from *Manglietia*. The reference genome used in this study was the chloroplast genome of *M. aromatica*1.

Sequence divergence among the twenty-two *Manglietia* plastomes was compared by aligning them with annotated *M. aromatic* plastomes as a reference using mVISTA. Sequence alignment revealed high sequence similarity across all twenty-two *Manglietia* plastomes without any observed rearrangement, suggesting their high conservation levels (Figure 2 and Figure 3). Whole-genome alignment indicated that non-coding regions displayed greater sequence variations (orange-colored bars) than protein-coding regions (purple-colored bars). The IR regions exhibited a higher degree of conservatism compared to the LSC and SSC regions. The intergenic spacer regions (IGS), such as *trnH-GUG*-*psbA*, *trnK-UUU*-*rps16*, *rps16-atpA*, *atpF-atpI*, *rpoB-psbD*, *psbC-psaB*, *psaA-ycf3*, *rps4-ndhJ*, *ndhC*-*atpE*, *atpB-rps12*, *rps7*-*ndhB*, and *ndhB-ycf15*, exhibited highly divergent non-coding regions in these chloroplast genomes. In contrast to the other genes, all the ribosomal RNA genes were highly conserved.

**Figure 4:**
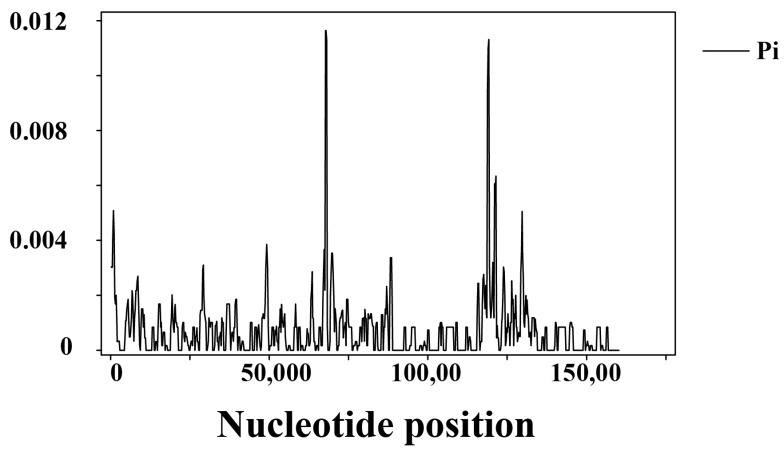
Comparison of the variation in nucleotide diversity (π) values across the twenty-two *Manglietia* plastomes. The vertical scale indicates nucleotide diversity (Pi) value and the horizontal axis represents sequence length.

To assess the divergence levels within different regions of these chloroplast genomes, nucleotide diversity (Pi) was measured by DnaSP within 600 bp windows. The SSC region exhibited the highest level of divergence (π = 0.00187), while the IR region showed the most conservative (π = 0.00022). Thirteen regions in the chloroplast genomes were identified as highly variable areas with Pi values exceeding 0.003. These regions include eight intergenic spacer regions (*trnH-GUG-psbA*, *rpoB-trnC-GCA*, *rps4-trnL-UAA*, *petA-psbJ*, *psbE-petL*, *rps3-rps19*, *ndhF-ccsA, ndhH-ycf1*) and five genes (*trnH-GUG*, *rps4*, *petA*, *ccsA*, *psaC*) within the coding regions (Figure 4).

We examined the expansion and contraction of the IR area between single-copy regions and pairs of IR regions for the twenty-two plastomes (Figure S1). The gene positions at four borders, JLB (LSC/IRb), JSB (IRb/SSC), JSA (SSC/IRa), and JLA (IRa/LSC), had almost identical types except that there were two situations for the JLB border. In *M. grandis* (NC_058271), the *rpl2* gene overlapped in the LSC/IRb region. Second, in other species, the *rpl2* gene was located in the IRb region and 56–60 bp away from the border, while in JSB junctional areas, the *ndhF* gene of all species was 61–105 bp away from border. JSA and JLA were both very conserved among the twenty-two plastomes. The *ycf1* gene straddles the boundary of JSA, with 5540–5648 bp in the IRa region. The distance of the junction between *trnH-GUG* and JLA ranges from 1 to 19 bp.

**Figure 5:**
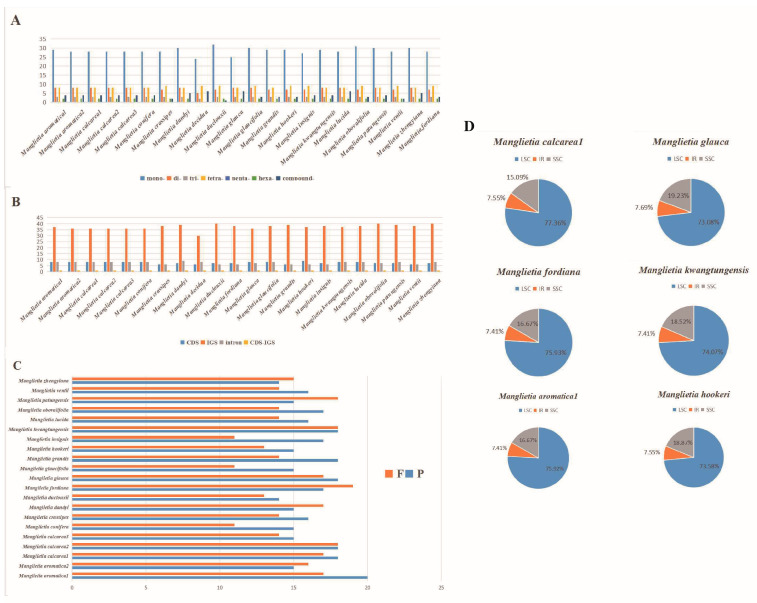
Analysis of repeat sequence maps in twenty-two plastomes derived from *Manglietia* species is presented. (**A**) Classification of SSRs based on repeat type, including momo-, di-, tri-, tetra-, penta-, hexa- and compound nucleotides. (**B**) Classification of SSRs in twenty-two plastomes, IGS, CDS, and CDS-IGS (**C**) Numbers of the four repeat types, F, P, R, and C. (**D**) SSRs locus distribution among three different regions.

The long repeat within the chloroplast genomes of *Manglietia* species was analyzed in this study, employing REPuter and Tandem Repeats Finder across twenty-two plastomes. A total of 1155 long repeats, of which 459 (39.74%) were tandem repeats, 342 (29.61%) forward repeats, and 354 palindromic repeats (30.65%), were identified in the genomes, and complement and reverse repeats were not found in *Manglietia* species (Table S5). The numbers of tandem repeats varied from 19 to 24, palindromic repeats from 13 to 19, and dispersed repeats from 11 to 19 (Figure 5C; Tables S2 and S3).

The plastomes of all twenty-two sequences were analyzed in this study, and a total of 51–56 SSRs were identified in each plastome, including six types (mono-, di-, tri-, tetra-, hexa-, and compound nucleotides), and “penta-” SSRs were not present. The details of all plastome SSRs are represented in Table S4. In all twenty-two chloroplast genomes, mononucleotides accounted for more than half (50.90%–59.26%), except for *M. glauca*. Among the different regions analyzed, the IGS region contained the largest number of SSRs (36–40), followed by 6–9 SSRs in both CDS and the coding sequence introns. Notably, A or T types dominated among mononucleotide SSRs, also exhibiting high richness within dinucleotide, trinucleotide, tetranucleotide, and hexanucleotide SSRs. In our results, fifty-four SSRs and sixty long repeat sequences were identified in the newly sequenced *M. aromatica*1. But in the published plastome of *M. aromatica*2, we identified fifty-three SSRs and forty-nine long repeat sequences. In the three samples of *M. calcarea,* 50, 59, and 59 long repeat sequences were identified, respectively (Figure 5A,B,D; Tables S3 and S5).

### 3.3. Codon Usage Bias Analysis

Codon usage bias is a fundamental genomic feature to provide crucial insights into species evolution. In total, 51 protein-coding genes were identified across the 22 chloroplast genomes analyzed. The GC content of these protein-coding genes ranged from 38.84% to 38.92%, with minimal variation observed in GC1, GC2, and GC3, all of which were below 42%. These findings also indicate a high abundance of A/T bases in the 22 *Manglietia* plastomes, particularly at the third codon position (Table 3). When examining codon usage bias through multi-species analysis, the effective number of codons (ENc) is commonly employed to quantify deviations from random selection and assess genome- or gene-specific biases. ENc values typically range between 20 and 61 [83]. Notably, lower ENc values suggest significant codon usage bias within a species’ genome or gene [84]. According to a previous study, when the ENc value is less than or equal to 35, it can be inferred that the species genome or gene codon usage bias is significant. In our study, the ENc values for all 22 *Manglietia* plastomes ranged from 50.34 to 50.97 (Table 3), significantly exceeding the threshold value of 35 and indicating weak codon usage bias within these plastomes. There were slight differences in the ENc, GC, GC1, and GC2 values between the two plastomes of *M. aromatica*. Three samples of *M. calcarea* also had little difference.

**Figure 6:**
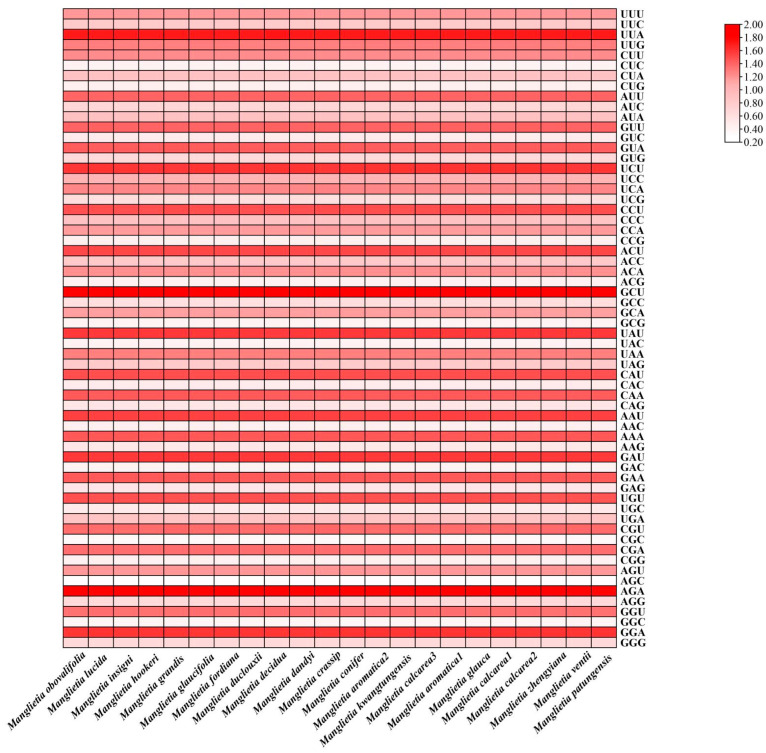
The RSCU of 22 plastomes from *Manglietia* species. Color variation closely relates to the RSCU value size.

The RSCU value denotes the ratio between the observed frequency of a codon’s usage and its theoretical expected frequency, serving as a crucial parameter for quantifying codon usage bias in scientific research. The terminator codons ATG and TGG were removed in this analysis, because terminator codons are not involved in encoding amino acids, and ATG and TGG only encode methionine and tryptophan without using bias. Overall, 51 protein-coding genes were encoded in 64 kinds of codons. Among them, 30 codons exhibited an RSCU value greater than 1, with 28 of these codons having A/T as their third bases. This indicates that high-frequency codons are more inclined to use A/T endings. There were 29 low-frequency codons in RSCU < 1, and 27 codons ending in G/C at the third base, accounting for 93.10%, indicating that the frequency of codons ending in G/C in the chloroplast genome is low. Among the synonymous codons of plastomes, GCY encoding alanine has the highest RSCU value, followed by UUA encoding isoleucine (Figure 6).

### 3.4. Phylogenetic Analysis

To ascertain the phylogenetic positions of *Manglietia* species and elucidate their evolutionary relationships, we constructed phylogenetic trees utilizing 77 protein-coding genes through the maximum likelihood (ML) and Bayesian inference (BI) methods. We combined 54 published chloroplast genomes of Magnoliaceae with five newly sequenced genomes, and trimmed some poor-alignment regions using Gblocks v 0.91b software. The aligned matrix of the CDS_GB dataset extracted by Geneious v11.1.5 showed a length of 68,520 bp in the 77 protein-coding region. For ML analysis, the best-fit model for the CDS_GB dataset was K3Pu + F + I and for BI analysis was GTR + F + I, as estimated by IQ-TREE.

**Figure 7:**
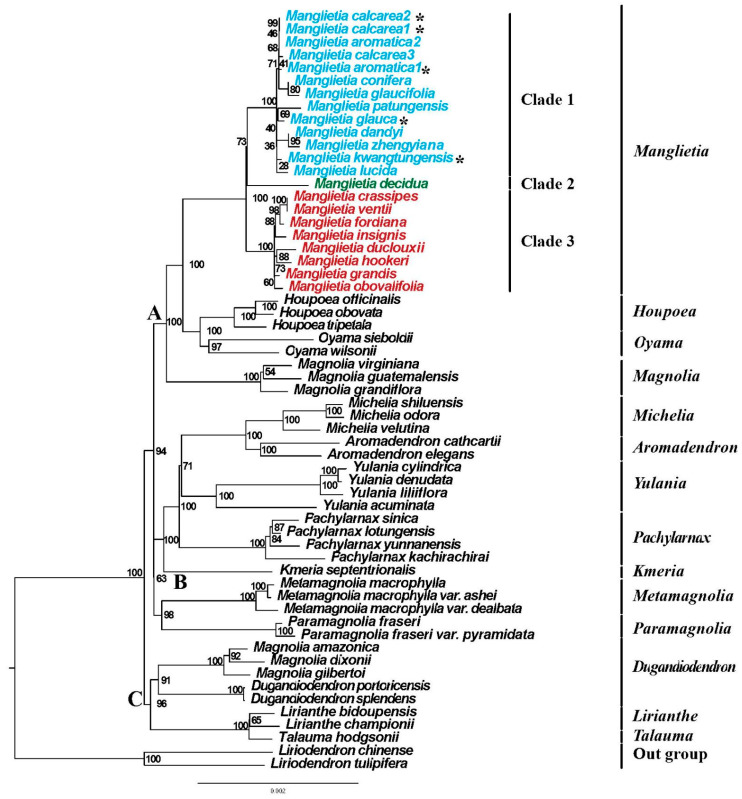
Maximum likelihood analysis based on a combined data matrix of 77 plastid genes for 59 species. The ”*” indicates our new sequences. The numbers indicate the phylogenetic support values from maximum likelihood with 100% bootstrap support (BS).

A phylogram of the maximum likelihood (ML) tree, displaying the support values at the nodes, is depicted in Figure 7. With *Liriodendron* as the outgroup, the members of Magnolioideae were divided into three major strongly supported groups (Figure 7A–C), each with ML bootstrap values (BS) of 94 and 100. Among them, the genera *Manglietia*, *Houpoea*, *Oyama*, and *Magnolia* were clustered into group A with strong support, the genera *Michelia*, *Aromadendron*, *Yulania*, *Pachylarnax*, *Kmeria*, *Metamagnolia*, and *Paramagnolia* were clustered into group B, and group C contained other genera. Our phylogenetic analysis strongly supports the result that there are 14 major clades within the subfamily Magnolioildeae that monophyletic.

The monophyly of the genus *Manglietia* was strongly supported in our study with high bootstrap support (BS = 100, PP = 1). The phylogenetic analysis revealed three distinct clades within the genus, namely clade 1, clade 2, and clade 3 (Figure 7 and Figure S2). Among them, clade 1 comprises *M. aromatica*, *M. calcarea*, *M. conifera*, *M. patungensis*, *M. glaucifolia*, *M. glauca*, *M. dandyi*, *M. zhengyiana*, *M. kwangtungensis*, and *M. lucida*; clade 2 just contains *M. decidua*; and clade 3 includes eight species: *M. crassipes*, *M. ventii*, *M. fordiana*, *M. insignis*, *M. duclouxii*, *M. hookeri*, *M. grandis*, and *M. obovalifolia*. All five newly sequenced plastomes are in clade 1. *M. glauca* and *M. patungensis* clustered into one branch with moderate support (BS = 69). *M. kwangtungensis* was sister to *M. lucida* and located at the base of clade 1 with weak support (BS = 28). The new sequences of *M. calcarea* were clustered into a group (BS = 99), and then, were found to be sisters to the published *M. aromatica*2 (BS = 46). However, our newly sequenced *M. aromatica*1 was clustered with the published *M. calcarea*3 with weak support (BS = 41), indicating that *M. calcarea* and *M. aromatica* became paraphyletic. Clade 3 can be classified into two distinct lineages, which have strong support (BS = 100).

### 3.5. Adaptive Evolution Analysis

The analysis of synonymous (Ks) and non-synonymous (Ka) substitution rates was conducted using a total of 51 protein-coding genes from all 22 plastomes of *Manglietia*. Twenty-two plastomes were compared in pairs, yielding a total of 231 results per gene. In our results, Ka and Ks were only found in some genes. In 12 genes (*ccsA*, *ndhE*, *petA*, *psaA*, *rpl20*, *rbcL*, *psbC*, *ycf4*, *ycf2*, *rps18*, *rpl14*, and *rpoC1*), Ka and Ks were identified only in clade 2 and clade 3, and these protein-coding genes had relatively low average values (Ka = 0.0019, Ks = 0.0071, Ka/Ks = 0.1322). Among *rps3* and *rpoC2*, only the species of Section 1 had Ka/Ks rates (Ka = 0.0016, Ks = 0.0049, Ka/Ks = 0.6036). In five genes (*accD*, *ndhH*, *rpl20*, *rpl2*, and *matK*), only Section 2 had Ka/Ks rates (Ka = 0.0009, Ks = 0.0054, Ka/Ks = 0.1629).

## 4. Discussion

### 4.1. Plastome Variation

In this study, we reported the plastomes of five individuals from four *Manglietia* species and compared them with those of 17 other *Manglietia* species to enhance our understanding of genome organization and molecular evolution of the Magnoliaceae family. The plastomes of most terrestrial plants exhibit highly conserved characteristics, including similar gene content and organization across different plant lineages [85]. All newly assembled *Manglietia* plastomes display the typical quadripartite structure observed in photosynthetic angiosperm plastomes, showing no notable distinctions compared to previously published chloroplast genomes within Magnoliaceae. The GC content of the twenty-two *Manglietia* plastomes analyzed in our study remains consistent at 39.3%, with the exception of *M. calearea*2 which has a slightly lower GC content of 39.0%. The higher GC content detected in the IR regions can be attributed to the presence of four copies of GC rRNA genes (*rrn16*, *rrn23*, *rrn4.5*, and *rrn5*) that are clustered in these regions, a common phenomenon in various plant species [86,87,88]. Compared with the GC content of the plastomes of other angiosperms, *Manglietia* has a higher GC content. The GC content is anticipated to exert a substantial influence on genome functioning and species ecology. It is postulated that higher GC content may be associated with enhanced adaptability to seasonally arid environments or cold regions, which are characteristic of a temperate climate for angiosperm species [89]. The plastome of five new sequences of *Manglietia* ranges from 157,093 bp (*M. calcarea*2) to 160,493 bp (*M. kwangtungensis*). It is similar in size to the plastomes of other previously published Magnoliaceae species [21,90]. The genome size of *M. calcarea*2 is approximately 3000 bp shorter than that of the other two samples, primarily due to a loss in the IR region encompassing the *rrn16-trnI-GAU* sequence. But another *M. calcarea* (*M. calcarea*1 from Dongduo) assembled complete sequence length is 160,446 bp, and that of the published *M. calcarea* (*M. calcarea*3) sequence is 160,027 bp. Comparing the plastomes between the two *M. aromatica* sequences, the new sequence is longer than the published plastome, mainly due to the difference of 213 bp in the LSC. The deletion of chloroplast genomes also leads to differences in the repeat sequence analysis and the codon usage bias analysis.

The genomics of the 22 *Manglietia* plastomes were compared using mVISTA and Mauve software. The genomics of 22 *Manglietia* chloroplast genomes exhibit a high degree of similarity, with significantly higher sequence variation observed in the non-coding and single-copy regions compared to the coding and IR regions. The borders of JLB are relatively conserved among angiosperm plastomes, primarily located within the *rps19* and *rpl2* genes. Our analysis reveals that *M. grandis* has the most pronounced contraction of the IR region, accompanied by an expansion of the *rpl2* gene at the JLB boundary by 308 bp, while the other 21 sequences have their *rpl2* genes situated within the IR region. This finding is consistent with a previous study on other *Manglietia* species [91]. It should be noted that modifications in IR boundaries can result in alterations in plastome size [92]. Notably, significant expansions and contractions have been reported in other plants, like *Pelargonium transcaalense*, which possesses a plastome size of 242,575 bp with an IR region spanning 87,724 bp [93]. However, the underlying mechanism governing expansion and contraction events within the plastid genome’s IR region remains unclear.

The coding regions and conserved sequences of the plastome have been extensively utilized for phylogenetic inferences at higher taxonomic levels, such as family or genus [94]. Plastomes serve as valuable resources for identifying mutational hotspots across different lineages and are employed in intraspecies discrimination and species-level phylogenetic studies [95,96]. However, certain plastid DNA fragments currently used in Magnoliaceae, including the *trnK* intron containing *matK*, *trnH-psbA*, *atpB-rbcL*, *rbcL*, the *trnL* intron, *trnL-trnF*, and *ndhF*, fail to provide sufficient phylogenetic signals required for establishing high-resolution relationships among related taxa. This limitation is particularly evident when classifying infrageneric taxa with uncertain taxonomic status [17,18]. To address this issue comprehensively within a *Manglietia* genus-specific context while maintaining a species-level focus on phylogenetic studies of *Manglietia* species groups, our alignment identifies the top 13 regions that exhibit the highest degree of genetic variability.

Molecular markers based on DNA polymorphisms, such as SSR, have emerged as valuable genetic resources widely employed for assessing genetic diversity and deducing molecular phylogenetics [97]. Simple sequence repeats (SSRs) are short (1–6 bp) repeat motifs that are tandemly repeated varying numbers of times [98]. SSRs can provide interspecific polymorphisms, making them effective markers in population genetic analysis. In the present study, a total of 1252 SSRs were identified across the 22 plastomes (Table S4); however, further experiments are required to verify their effectiveness. Mononucleotide SSRs represent the most prevalent motifs and occupy the largest portion among all SSR types. Tri- and hexanucleotide SSRs occur at much lower frequencies (Figure 5A). Pentanucleotide SSRs were not found in any of the twenty-two plastomes examined. Additionally, most of the detected SSRs consist of A/T repeat units, which may contribute to the high AT richness observed in these chloroplast genomes. Further statistical analysis reveals that these polymorphic variations are not evenly distributed throughout the plastomes. Compared to both single-copy regions and inverted repeat regions, large single-copy regions contain a higher number of SSRs. As expected, sequence variations primarily occur within the LSC region and non-coding regions such as intergenic spacer regions (Figure 5B,D). Similar findings have been reported in other angiosperm plastid genomes [99,100].

### 4.2. Phylogenetic Analysis

Chloroplast genomes have been utilized in phylogenetic analyses due to their nonrecombinant and uniparentally inherited nature, as well as their comparatively slower evolutionary rates compared to nuclear and mitochondrial genomes [101,102]. The plastome regions of *matK* and *ndhF* have demonstrated remarkable success as genetic markers within the Magnoliaceae family [17]. However, the limited number of loci used in phylogenetic inference may lack sufficient power when closely related species are being considered. Consequently, there is a growing preference for conducting phylogenetic analyses based on comprehensive plastome datasets, necessitating comparative genomic studies involving a larger number of plastome sequences.

The phylogenetic analysis based on the protein-coding regions derived from chloroplast genomes also strongly support that the classification of two subfamilies Liriodendroideae and Magnolioideae, as proposed by Law [7,8]. Liriodendroideae include a single genus, *Liriodndron*, with only two species disjunctly distributed in Eastern Asia and eastern North America [103]. However, the classification of Magnolioideae has been highly debated for a long time. Some authors [10,21,24,31] argued that a single expanded genus *Magnolia* would be accepted and it may be divided into 9–15 sections based on morphology or molecular evidence, while many botanists [3,4,5,6,9,11] prefered to maintain a narrow generic delimitation and recognize up to 16 genera within Magnolioideae. Our phylogenetic analysis strongly supports 14 major clades within Magnolioideae, which correspond to the 14 genera proposed by Sima and Lu [3], indicating that maintaining a narrow generic concept would be better to present the evolutionary tendency within Magnolioideae. *Manglietia* has been widely accepted as an independent genus for a long time [3,6,7,8,11]. Meanwhile, the monophyly of *Manglietia* has been supported by all previous phylogenetic studies [14,15,17,18,21,22,24,104]. Previous phylogenetic analysis using nuclear data [24] indicated that *Manglietia* and *Houpoea* (sect. *Rytidospermum*) formed sister groups, and then, they were found to be sisters to *Oyama* (sect. *Oyama*). However, in our study, *Houpoea* and *Oyama* are found to be sister groups with a strong support (BS = 100), and then, they are found to be sisters to *Manglietia* (BS = 100). Discordance between nuclear and organellar phylogenies is commonly observed across the plant tree [105,106]. This incongruence in tree topology may arise from various biological phenomena, including gene duplication, horizontal gene transfer, incomplete lineage sorting, or gene flow [107,108]. In our study, due to insufficient data and under-sampling, we were unable to further analyze this phylogenetic conflict.

**Figure 8:**
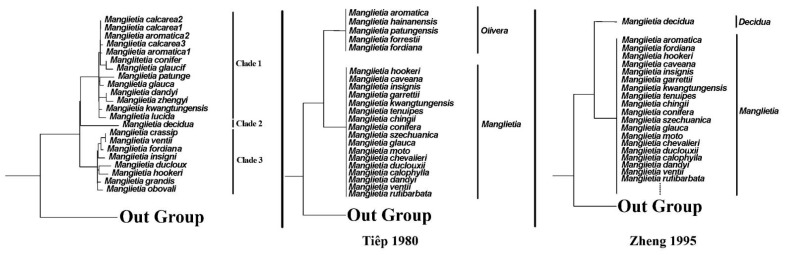
The different infrageneric classifications of *Manglietia*. The three phylogenetic trees represent the result of this study, the result of Tiěp based on morphological characteristics, and the result of Zheng based on deciduous habits [36,37].

The infra-generic classification of *Manglietia* is still unclear. Tiep [36] and Zheng [37] attempted to propose infrageneric classifications (Figure 8), but their classifications have never been adopted by other authors. Based on the scale of the style and carpel length, Tiep [36] divided the genus *Manglietia* into two sections, sect. *Manglietia* and sect. *Olivera*. However, our results indicate that both sect. *Manglietia* and sect. *Olivera* are not monophyletic. When they merged the genus *Sinomanglietia* with *Manglietia*, Zheng et al. [37] divided the genus into two sections, sect. *Decidua* and sect. *Manglietia*. Sect. *Decidua*, originally described as the genus *Sinomanglietia* includes a single species *M. decidua*, with a deciduous habit, while all other species with evergreen habits were placed in sect. *Manglietia*. However, in our studies, *M. decidua*, a representative of sect. *Decidua*, is nested within sect. *Manglietia*. It is suggested by the results of Wang et al. [21], based on 86 whole chloroplast genomes, that *M. decidua* is the sister of all other sampled *Manglietia*. In the present study, the CDS_GB dataset divided twenty-two plastomes from *Manglietia* into three clades (Figure 7 and Figure S2) with bootstrap values (BS) of 73 and 100 and BI posterior probabilities (PP) of 0.67 and 1.00. Clade 1 consists of *M. aromatica*, *M. calcarea*, *M. conifera*, *M. patungensis*, *M. glaucifolia*, *M. glauca*, *M. dandyi*, *M. zhengyiana*, *M. kwangtungensis*, and *M. lucida*. Clade 2 includes a single species, *M. decidua*, with a deciduous habit. Clade 3 comprises *M. crassipes*, *M. ventii*, *M. fordiana*, *M. insignis*, *M. duclouxii*, *M. hookeri*, *M. grandis*, and *M. obovalifolia*. This implies that the genus may be divided into three sections, but further studies are necessary. The relationships among most *Manglietia* species nodes have strong support values (bootstrap support value > 70 and Bayesian posterior probability > 0.90), while a few nodes are weak (bootstrap support value < 50), and the phylogenetic relationships of some species are still unclear. As for the plastomes of the five newly sequenced species in this study, *M. kwangtungensis* is sister to *M. lucida* and located at the base of clade 1 with weak support (BS = 28). The sister relationship between *M. glauca* and *M. patungensis* also has weak support (BS = 69, PP = 0.94); this may be due to incomplete taxon sampling. The treatment of *M. calcarea* has been controversial in the past. Chen and Nooteboom [35] and Kumar [109] treated it as a variety (*M. fordiana* var. *calcarea* and *M. fordiana* var. *calcarea*, respectively). Xia et al. [11] reinstated it as independent species. Later, Sima et al. [59] considered it to be closely related to *M. aromatica* and treated it as a variety, *M. aromatica* var. *calcarea*. Our results show that *M. calcarea* is isolated with *M. fordiana*, each located in different clades, clade 1 and clade 3, respectively. *M. fordiana* is located in clade 3, and sister to *M. crassipes* and *M. ventii*, with strong support (BS = 98, PP = 1.00). This implies that it is not appropriate to treat *M. calcarea* as a variety of *M. fordiana*. Morphologically, *M. calcarea* differs from *M. fordiana* in that it has 12–16 carpels and mature carpels dehiscing along the dorsal sutures. However, *M. calcarea* and *M. aromatica* are clustered into a group, and the previously published *M. aromatica*2 is nested within *M. calcarea*, and then, forms sister groups with the currently sequenced *M. aromatica*1. The sequence difference between the two species is only 305 bp, and the main difference is the loss of bases in the intergenic spacer (Figure 2). Therefore, the affinity between *M. calcarea* and *M. aromatica* is supported by our studies. But this suggests that *M. calcarea* and *M. aromatica* are not monophyletic. It seems that *M. calcarea* may be considered to be different from *M. aromatica* as its leaves are coriaceous, rigid, and rounded with an acumen 6–14 mm long at the apex, and it has 9 tepal 9 12–16 carpels, while in the latter, the leaves are thinly coriaceous to coriaceous, mucronate to acuminate at the apex, and it has 11–12 tepals and 29–39 carpels [59]. *M. calcarea* is recorded from the neighboring areas between N Guangxi (Huanjiang) and S Guizhou (Libo and Dushan), while *M. aromatica* is distributed in W Guangxi, SW Guizhou, and Yunnan Province. Sometimes, *M. calcarea* is identified as *M. aromatica* in the literature [110,111]. At the moment, we cannot examine the voucher of previously sampled *M. aromatica*2, and its identification needs be confirmed. It seems that our studies suggest merging *M. calcarea* with *M. aromatica*. However, the final determination on the species *M. calcarea* and *M. aromatica* needs be further studied to include more samples in their distribution ranges.

## 5. Conclusions

In the present study, we assembled and analyzed five new complete or nearly complete plastome sequences of *Manglietia* species and conducted the first comparative analysis with other *Manglietia* species. The annotation and comparison within *Manglietia* species revealed conservation of the gene sequence, GC content, and genomic composition. Additionally, we identified repeated sequences, 51–56 microsatellites, and 13 highly mutational hotspot regions in the *Manglietia* plastome. These findings contribute to our understanding of the adaptations of *Manglietia* species to limestone environments. Furthermore, they provide valuable genomic resources and potential markers for future studies on species identification and speciation within this genus. This study sheds light on the phylogenetic relationships and adaptive evolution of *Manglietia*. The genus may be divided into three sections, and the previously proposed infrageneric classifications are not supported by our studies. The close affinity between *M. calcarea* and *M. aromatica* is supported, but their taxonomic treatment needs to be further studied.

## Figures and Tables

**Table 1:** The basic characteristics of twenty-two plastomes from *Manglietia* species.

Species	Accession Number	Length (GC%)	LSC (GC%)	IR (GC%)	SSC (GC%)	Gene	CDS	tRNA	rRNA
*Manglietia aromatica*1	PP386161	160,446 (39.3%)	88,300 (38.0%)	26,677 (43.1%)	18,792 (34.3%)	113	79	30	4
*Manglietia aromatica*2	NC_037000	160,062 (39.3%)	88,087 (38.0%)	26,572 (43.2%)	18,831 (34.2%)	113	79	30	4
*Manglietia calcarea1*	PP386158	160,446 (39.3%)	88,297 (38.0%)	26,572 (43.2%)	19,005 (34.3%)	112	79	29	4
*Manglietia calcarea2*	PP386157	157,093 (39.0%)	88,298 (38.0%)	24,991 (42.5%)	18,813 (34.2%)	110	79	28	3
*Manglietia calcarea*3	MF990562	160,027 (39.3%)	88,088 (38.0%)	26,572 (43.2%)	18,795 (34.2%)	113	79	30	4
*Manglietia conifera*	NC_037001	159,973 (39.3%)	88,088 (38.0%)	26,572 (43.2%)	18,741 (34.3%)	113	79	30	4
*Manglietia crassipes*	NC_058270	159,901 (39.3%)	87,959 (38.0%)	26,571 (43.2%)	18,800 (34.2%)	113	79	30	4
*Manglietia dandyi*	NC_037004	160,077 (39.3%)	88,095 (38.0%)	26,572 (43.2%)	18,838 (34.2%)	113	79	30	4
*Manglietia decidua*	OQ773531	160,151 (39.3%)	88,198 (37.9%)	26,566 (43.2%)	18,821 (34.2%)	113	79	30	4
*Manglietia duclouxii*	NC_037002	160,055 (39.3%)	88,118 (38.0%)	26,574 (43.2%)	18,789 (34.3%)	113	79	30	4
*Manglietia fordian* *a*	MN515039	160,074 (39.3%)	88,100 (37.9%)	26,576 (43.3%)	18,822 (34.3%)	113	79	30	4
*Manglietia glauca*	PP386159	160,459 (39.3%)	88,298 (38.0%)	26,572 (43.2%)	19,017 (34.3%)	113	79	30	4
*Manglietia glaucifolia*	MF990565	160,059 (39.3%)	88,094 (38.0%)	26,581 (43.2%)	18,803 (34.3%)	113	79	30	4
*Manglietia grandis*	NC_058271	160,008 (39.3%)	88,791 (38.0%)	26,207 (43.2%)	18,803 (34.3%)	113	79	30	4
*Manglietia hookeri*	MW415420	160,035 (39.3%)	88,098 (38.0%)	26,576 (43.2%)	18,785 (34.3%)	113	79	30	4
*Manglietia insignis*	MT654128	160,051 (39.3%)	88,139 (38.0%)	26,583 (43.2%)	18,746 (34.3%)	113	79	30	4
*Manglietia kwangtungensis*	PP386160	160,493 (39.3%)	88,319 (37.9%)	26,572 (43.2%)	19,030 (34.3%)	112	79	29	4
*Manglietia lucida*	MT682867	160,134 (39.3%)	88,119 (38.0%)	26,595 (43.2%)	18,825 (34.2%)	113	79	30	4
*Manglietia obovalifolia*	NC_058551	160,073 (39.3%)	88,113 (38.0%)	26,576 (43.2%)	18,808 (34.3%)	113	79	30	4
*Manglietia patungensis*	OP689708	160,139 (39.3%)	88,102 (38.0%)	26,572 (43.2%)	18,893 (34.3%)	111	79	28	4
*Manglietia ventii*	NC_058272	159,950 (39.3%)	88,008 (38.0%)	26,571 (43.2%)	18,800 (34.2%)	113	79	30	4
*Manglietia zhengyiana*	OP689709	160,058 (39.3%)	88,094 (38.0%)	26,572 (43.2%)	18,820 (34.2%)	111	79	28	4

Note: GC, content of guanine–cytosine; CDS, protein-coding gene.

**Table 2:** List of genes encoded by twenty-two plastomes of *Manglietia* species.

Classification	Genes
Genetic apparatus	
Large ribosomal subunits	*rpl2 rpl14 rpl16 rpl20 rpl22 rpl23 rpl32 rpl33 rpl36*
Small ribosomal subunits	*rps2 rps3 rps4 rps7 rps8 rps11 rps12 rps14 rps15 rps16 rps18 rps19*
RNA polymerase subunits	*rpoA rpoB rpoC1 rpoC2*
DNA-dependent RNA Polymerase protease	*clpP*
Maturase	*matK*
Ribosomal RNAs	*rrn4.5 rrn5 rrn16 rrn23*
Transfer RNAs	*trnA-UGC trnC-GCA trnD-GUC trnE-UUC trnF-GAA trnfM-CAU* *trnG-GCC trnG-UCC trnH-GUG trnI-CAU trnI-GAU trnK-UUU* *trnL-CAA trnL-UAA trnL-UAG trnM-CAU trnN-GUU trnP-UGG* *trnQ-UUG trnR-ACG trnR-UCU trnS-GCU trnS-GCU trnS-GGA* *trnS-GGA trnS-UGA trnT-GGU trnT-GGU trnT-UGU trnT-UGU* *trnV-GAC trnV-UAC trnW-CCA trnY-GUA*
Photosystem I	*psaA psaB psaC psaI psaJ ycf3 ycf4*
Photosystem II	*psbA psbB psbC psbD psbE psbF psbH psbI psbJ psbK psbL psbM psbN* *psbT psbZ*
NAD(P)H dehydrogenase complex	*ndhA ndhB ndhC ndhD ndhE ndhF ndhG ndhH ndhI ndhJ ndhK*
F-type ATP synthase	*atpA atpB atpE atpF atpH atpI*
Cytochrome b6/f complex	*petA petB petD petG petL petN*
Inner membrane protein	*cemA*
Cytochrome C biogenesis protein	*ccsA*
Large subunit of Rubisco	*rbcL*
Subunit of acetyl-CoA-carboxylase	*accD*
Translation initiation factor	*infA*
Function uncertain	*ycf1 ycf2 ycf15 ycf68*

**Table 3:** Codon features of chloroplast genomes of 22 plastomes from *Manglietia* plant species.

Species	ENc	GC3s	GC	GC1	GC2	GC3
*Manglietia aromatica*1	50.6	0.284	38.87	38.64	39.49	38.48
*Manglietia aromatica*2	50.59	0.284	38.86	38.63	39.48	38.48
*Manglietia calcarea*1	50.59	0.284	38.87	36.5	40.49	39.63
*Manglietia calcarea*2	50.59	0.284	38.87	39.06	39.84	37.72
*Manglietia calcarea*3	50.59	0.284	38.86	36.49	40.48	39.62
*Manglietia conifera*	50.57	0.284	38.87	36.5	40.49	39.61
*Manglietia crassipes*	50.53	0.283	38.85	39.14	39.13	38.27
*Manglietia dandyi*	50.6	0.284	38.87	39.72	39.41	37.46
*Manglietia decidua*	50.49	0.283	38.92	36.91	40.83	39.02
*Manglietia duclouxii*	50.57	0.284	38.87	39.5	38.44	38.68
*Manglietia fordiana*	50.58	0.284	38.84	37.88	39.5	39.14
*Manglietia glauca*	50.6	0.284	38.88	38.24	40.44	37.96
*Manglietia glaucifolia*	50.57	0.284	38.86	39.95	36.27	40.37
*Manglietia grandis*	50.54	0.283	38.85	39.93	37.06	39.56
*Manglietia hookeri*	50.56	0.284	38.86	39.35	39.13	38.1
*Manglietia insignis*	50.56	0.284	38.88	38.32	38.97	39.34
*Manglietia kwangtungensis*	50.59	0.284	38.88	38.47	37.94	40.22
*Manglietia lucida*	50.58	0.284	38.87	36.96	40.8	38.86
*Manglietia obovalifolia*	50.57	0.284	38.84	40.43	34.21	41.89
*Manglietia patungensis*	50.6	0.284	38.85	41.63	38.39	36.53
*Manglietia ventii*	50.53	0.283	38.85	39.14	39.13	38.27
*Manglietia zhengyiana*	50.58	0.284	38.85	39.67	39.53	37.35

Note: ENc, effective number of codons; GC, content of guanine–cytosine; GC3s, probability that the third base of the codon appears G/C; GC1/2/3, GC content of the first, second, and third codon bases.

## Data Availability

The chloroplast genomes of *Manglietia calcarea*1, *Manglietia calcarea*2, *Manglietia glauca*, *Manglietia kwangtungensis*, and *Manglietia aromatica*1 have been deposited in the NCBI database with the accession numbers PP386157, PP386158, PP386159, PP386160, and PP386161, respectively.

## Supplementary Materials

The following supporting information can be downloaded at: https://www.mdpi.com/article/10.3390/genes15040406/s1, Table S1: Information of five samples of *Manglietia* newly sequenced in this study; Table S2: Long repeat sequences in *Manglietia* plastomes; Table S3: Tandem repeat distribution in twenty-two *Manglietia* plastomes; Table S4: Simple sequence repeats distribution in twenty-two *Manglietia* plastomes; Table S5: Types and numbers of long repeats in the twenty-two *Manglietia* chloroplast genomes. Figure S1: Comparison of the borders of LSC, SSC, and IR regions in twenty-two complete chloroplast genomes. JLB (IRb /LSC), JSB (IRb/SSC), JSA (SSC/IRa) and JLA (IRa/LSC) denote the JSs between each corresponding region in the genome. Figure S2: Bayesian inference based on a combined data matrix of 77 plastid genes for 59 species.
