# Supplementary material for: Comparative Analysis of Chloroplast Genomes for the Genus Manglietia Blume (Magnoliaceae): Molecular Structure and Phylogenetic Evolution
Source: Genes (Basel). 2024 Mar 26;15(4):406. doi: 10.3390/genes15040406 (PMC11048997; doi:10.3390/genes15040406)
Supplement: Supplementary file 1 [file genes-15-00406-s001.zip › genes-2915740-supplementary-ST.pdf]

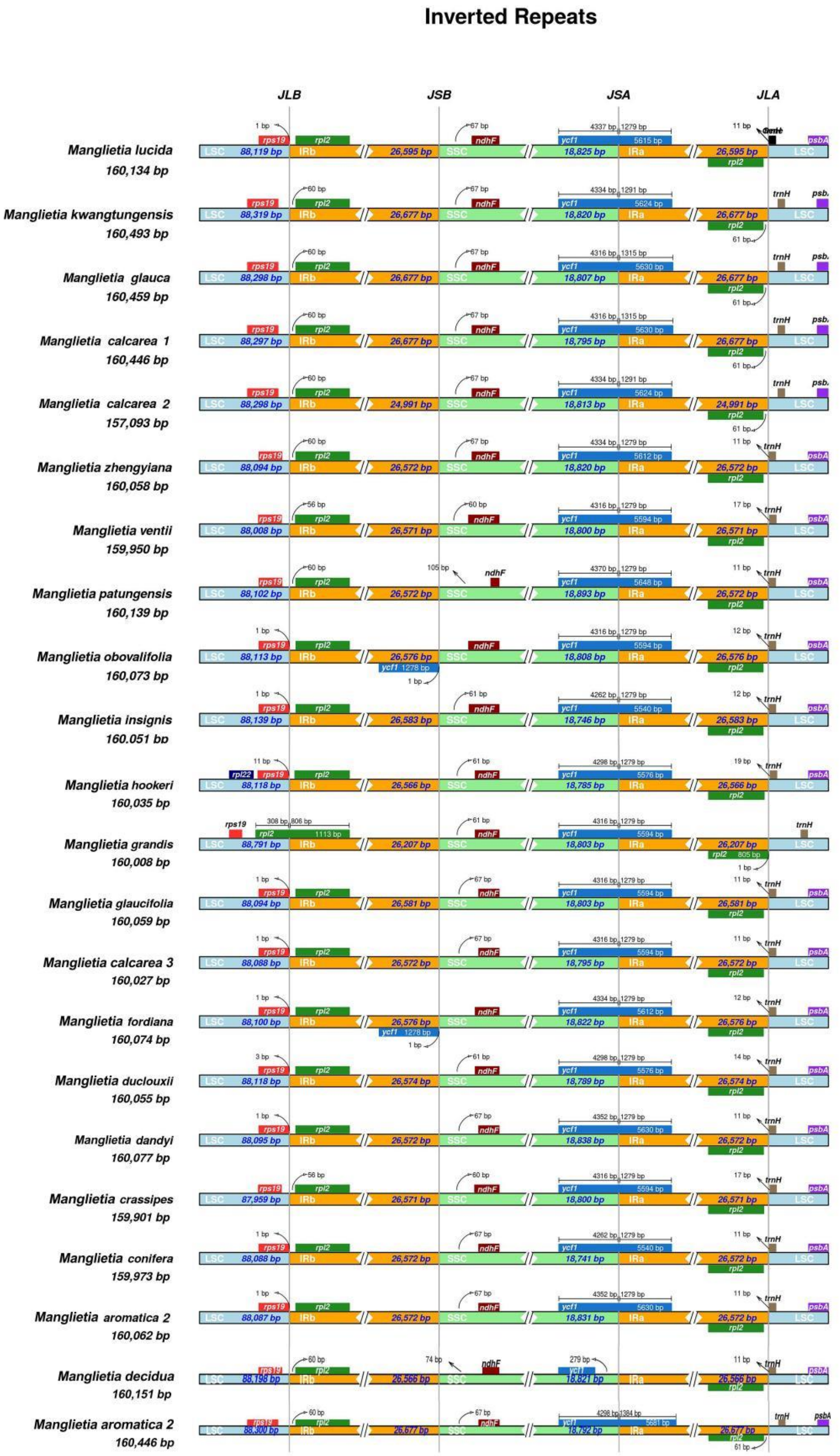

Figure S1. Comparison of the borders of LSC, SSC, and IR regions in twenty-two complete chloroplast genomes. JLB (IRb /LSC), JSB (IRb/SSC), JSA (SSC/IRa) and JLA (IRa/LSC) denote the JSs between each corresponding region in the genome.

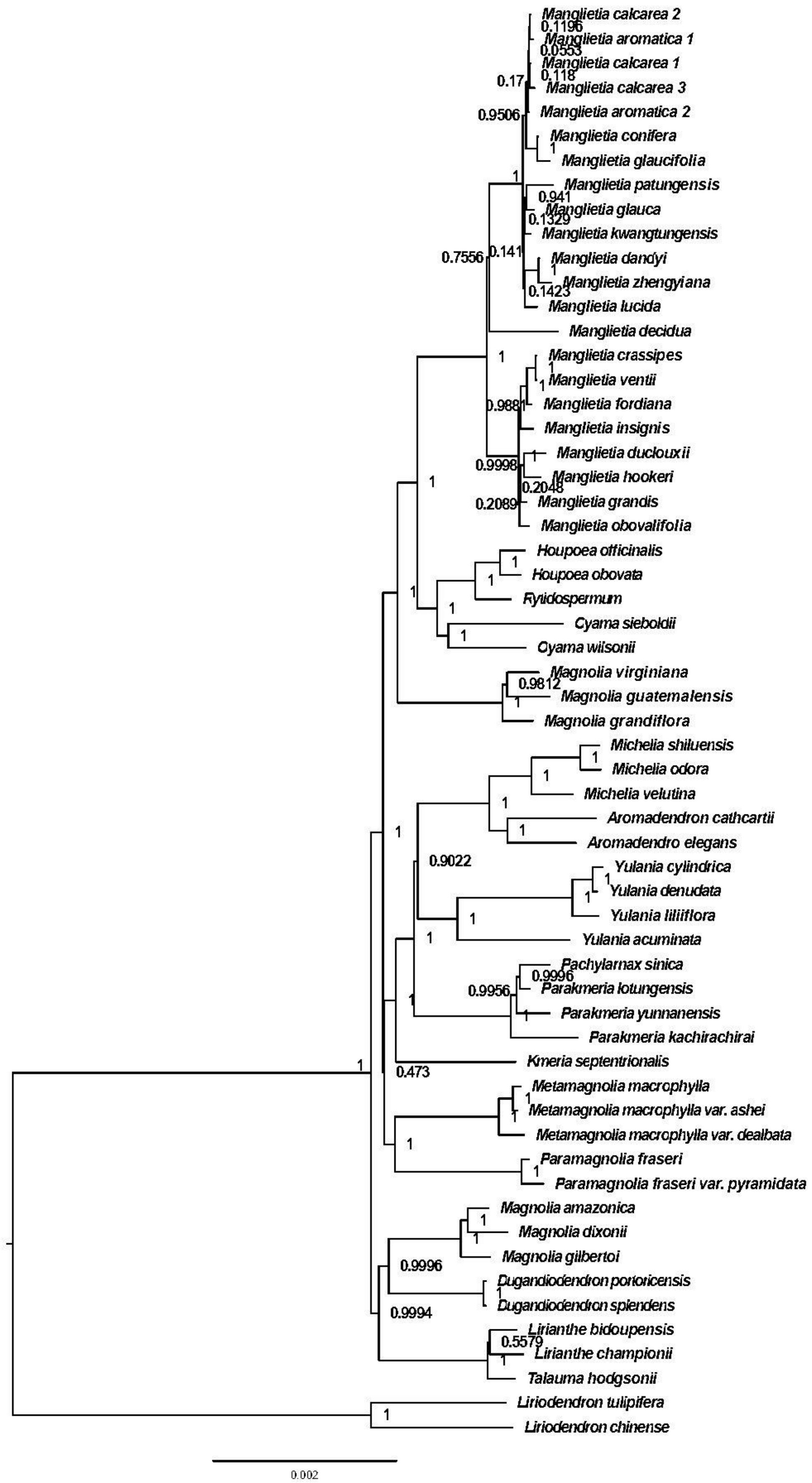

Figure S2. Bayesian inference based on a combined data matrix of 77 plastid genes for 59 species.
